# Complex Orthopaedic Trauma Is Shifting Away From Level I to Non–Level I Trauma Centers: An Analysis of the National Trauma Data Bank

**DOI:** 10.5435/JAAOSGlobal-D-22-00288

**Published:** 2023-02-07

**Authors:** Meir T. Marmor, Sarah Coufal, Philip M. Parel, Arash Rezaei, Saam Morshed

**Affiliations:** From the University of California San Francisco (UCSF), Orthopaedic Trauma Institute (OTI), Zuckerberg San Francisco General Hospital, San Francisco, CA (Dr. Marmor and Dr. Morshed); Department of Orthopaedic Surgery, the University of California Los Angeles (UCLA), Los Angeles, CA (Coufal); Department of Orthopaedic Surgery, the George Washington University School of Medicine and Health Sciences, Washington, DC (Mr. Parel); and the Department of Orthopaedic Surgery, University of Illinois Chicago, Chicago, IL (Dr. Rezaei).

## Abstract

An increasing number of fellowship-trained orthopaedic trauma surgeons are working in non–Level I centers. This study aimed to examine trends of management of complex orthopaedic trauma in Level I centers versus non–Level I centers and its potential effect on patient outcomes.

**Methods:**

Data from the National Trauma Data Bank from 2008 to 2017 were analyzed. Non–Level I to Level I center ratios for complex fractures and complication rates, median hours to procedure for time-sensitive fractures, and uninsured/underinsured rates of Level I and non–Level I centers were recorded.

**Results:**

Three hundred one thousand patients were included. A statistically significant downward trend was identified in the percent of all complex orthopaedic trauma at Level I centers and per-hospital likelihood of seeing a complex orthopaedic fracture in a Level I versus non–Level I hospital. Per-hospital complication rates were consistently lower in non–Level I hospitals after controlling for injury severity and payer mix. Time-sensitive fractures were treated earlier in non–Level I centers.

**Discussion:**

This study demonstrates a reduction of complex trauma treatment in Level I centers that did not translate to adverse effects on patient outcomes. Policymakers should notice this trend to ensure the continued quality of orthopaedic trauma training and maintenance of expertise in complex fracture management.

Level I trauma centers are the backbone of trauma systems in North America and worldwide. They provide the highest level of care to patients with trauma.^1-3^ They are not only primarily responsible for training the next generation of trauma caregivers, including orthopaedic trauma surgeons, but also have research roles, provide injury prevention education in their respective communities, and establish outreach programs to emergency providers. Per this role, Level I trauma centers are required to provide the highest quality of care for the most complex orthopaedic trauma. Recent years have shown an increase in fellowship-trained orthopaedic trauma surgeons.^4-6^ Because of the limited number of academic positions at Level I centers, many of these surgeons, who are specialty trained to treat complex musculoskeletal injuries, practice in Level II and III trauma centers, which lack the research, outreach, and injury prevention programs needed for Level I certification.^7^ Therefore, we hypothesized that there should be a trend of increasing management of complex orthopaedic trauma in non–Level I trauma centers without a detrimental effect on outcomes. In this study, we reviewed a national trauma registry to test for trends in the management of complex orthopaedic trauma in Level I trauma centers versus non–Level I trauma centers and its potential effect on patient outcomes.

## Methods

The RECORD guidelines for reporting of studies conducted using observational routinely collected health data were used to ensure proper reporting of methods, results, and discussion.^8^

### Setting

We queried the National Trauma Data Bank (NTDB) for patients treated for complex orthopaedic trauma. The NTDB is a deidentified trauma patient registry compiled and maintained by the American College of Surgeons (ACS). In 2017, the NTDB contained detailed data on over seven million records from over 900 registered US trauma centers.^9,10^ The data sets used for this project spanned over a decade from 2008 to 2017. Data from the NTDB files were imported and stored in a relational database. The data were read into pandas DataFrames inside a Jupyter notebook (Version 6.0.3, Jupyter, 2020) using Python language (Version 3.8.3, Python TM, 2020). Pandas is a Python data analysis library that is imported. Relevant variables were extracted, manipulated for analysis, and processed by both Python and Microsoft Excel (Version 16.41, Microsoft, 2020). This study was conducted in accordance with the approval exemption from the Institutional Review Board of the address of correspondence.

### Participants

Patients between 18 and 65 years of age who received treatment for complex orthopaedic trauma were included in the study. We defined complex orthopaedic trauma as a complex surgical fracture fixation or a time-sensitive orthopaedic fracture. The complex fracture fixations we considered for this study were nondiaphyseal (articular or periarticular), open or closed fractures, requiring surgical treatment by internal fixation (after open or closed reduction), excluding hip and distal radius fractures: proximal humerus, distal humerus, proximal tibia, distal tibia, and distal femur. Fractures of the pelvis, acetabulum, calcaneus, and talus were also considered complex. *International Classification of Diseases Revision 9* diagnostic codes and procedure codes were used for years 2008 to 2015, and *International Classification of Diseases Revision 10* diagnostic codes and procedure codes were used for years 2016 to 2017 (Table 1). Time-sensitive procedures included Open Reduction and Internal Fixation (ORIF) or Closed Reduction and Internal Fixation (CRIF) of the following fractures that require timely management: open tibia fractures, open femoral shaft fractures, open distal femur fractures, closed femoral shaft fractures, and open or closed femoral neck fractures in patients aged 50 years or younger (Table 1). A cutoff of 50 years of age was selected to conform to similar studies, which used 50 years as the distinction between younger and older patients.^11^ Combining both the diagnostic and procedure codes reduced the chance for misclassification. In 2007 to 2015, the principal procedure codes applied were ORIF and CRIF. For 2016 to 2017 the procedure codes listed under reposition and internal fixation using open and percutaneous approaches were applied.

**Table 1:** *ICD-9* and *-10* Diagnostic and Procedure Codes for Selected Complex Fractures and Time-Sensitive Fractures

Fracture Type	*ICD-9* Diagnostic Code	*ICD-10* Diagnostic Code		*ICD-9* Procedure Code	*ICD-10* Procedure Code
		Starts With	Ends With		
Complex fractures					
Open proximal or distal humerus	812.1-812.19; 812.5-812.59	S42.2 and S42.4	B or C	79.31	0PSC04Z, 0PSC06Z, 0PSC34Z, 0PSC36Z, 0PSD04Z, 0PSD06Z, 0PSD34Z, 0PSD36Z, 0PSF04Z, 0PSF06Z, 0PSF34Z, 0PSF36Z, 0PSG04Z, 0PSG06Z, 0PSG34Z, and 0PSG36Z
Closed proximal or distal humerus	812.1-812.19; 812.5-812.59	S42.2 and S42.4	A	79.31	
Open proximal or distal tibia	823.1, 824.1, and 823.9	S82.1 and S82.3	B or C	79.36	0QSG04Z, 0QSG06Z, 0QSG34Z, 0QSG36Z, 0QSH04Z, 0QSH06Z, 0QSH34Z, and 0QSH36Z
Closed proximal or distal tibia	823, 824, and 823.8	S82.1 and S82.3	A	79.36	
Open distal femur	821.3-821.39	S72.4	B or C	79.35 and 79.15	0QSB04Z,0QSB06Z, 0QSB34Z,0QSB36Z, 0QSC04Z,0QSC06Z, 0QSC34Z, and 0QSC36Z
Closed distal femur	821.0-821.29		A	79.35 and 79.15	
Open and closed pelvis and acetabulum	808-808.9	S32.2, S32.4, S32.5 S32.6, and S32.8	N/A	79.39	0QS204Z, 0QS234Z, 0QS304Z, 0QS334Z, 0QS404Z, 0QS434Z, 0QS504Z, and 0QS534Z
Closed calcaneus	825	S92.0	A	79.39	0QSL04Z, 0QSL34Z, 0QSM04Z, and 0QSM34Z
Closed talus	825.21	S92.1	A	79.39	
Time-sensitive fractures					
Open tibia (proximal, shaft, and distal)	812.1-812.19; 812.5-812.59	S42.2 and S42.4	B or C	79.16 and 79.36	0QSG04Z, 0QSG06Z, 0QSG34Z, 0QSG36Z, 0QSH04Z, 0QSH06Z, 0QSH34Z, and 0QSH36Z
Open femur (distal and shaft)	821.1-821.11; 821.3-821.39	S72.3 and S72.4	B or C	79.15 and 79.35	0QSB04Z,0QSB06Z, 0QSB34Z,0QSB36Z, 0QSC04Z,0QSC06Z, 0QSC34Z, 0QSC36Z, 0QS804Z, 0QS806Z, 0QS834Z, 0QS836, 0QS904Z, 0QS906Z, 0QS934Z, and 0QS936Z
Closed femur (shaft)	821.0-821.01	S72.3	A	79.35	0QS804Z, 0QS806Z, 0QS834Z, 0QS836Z, 0QS904Z, 0QS906Z, 0QS934Z, and 0QS936Z
Femoral neck (<50 years old)	820-820.19; 820.8-820.9	S72.0	N/A	79.35	0QS604Z, 0QS606Z, 0QS634Z, 0QS636Z, 0QS704Z, 0QS706Z, 0QS734Z, and 0QS736Z

### Variables

We recorded age, fracture diagnosis code, fracture procedure code, time to procedure, trauma center level, complications during the hospital stay, Injury Severity Scale (ISS), and payment type for each participant.

#### Trauma Center Level

Following the study objective, we grouped patients into Level I patients and non–Level I patients. The NTDB includes an ACS verification level and a state designation Level for each trauma center. These two levels could either match or differ for a single trauma center. When the state designation level and ACS verification level differed, the ACS verification level was used. If the trauma center was listed as not applicable in the ACS level, the state-designated level was used. We excluded records that had both the ACS and the state designation as not applicable. Non–Level I patients included patients in trauma centers listed as Level II-V.

#### Complications in the Initial Hospital Stay

The NTDB only records complications that occur during the initial hospital stay. We recorded the following nonorthopaedic complications: acute kidney injury, acute respiratory distress syndrome, deep vein thrombosis, myocardial infarction, organ/space surgical site infection, pulmonary embolism, stroke/cerebrovascular accident, unplanned intubation, unplanned return to the operating room, unplanned admission to the Intensive Care Unit, severe sepsis, catheter-associated urinary tract infection, central line-associated bloodstream infection, ventilator-associated pneumonia, severe sepsis, and death. The following orthopaedic complications were also recorded: deep surgical site infection, superficial surgical site infection, extremity compartment syndrome, and osteomyelitis. Having any number of complications for a single patient was considered a single complication event in the analysis.

#### Payment Type

We determined the payer mix of Level I and non–Level I hospitals by values entered as their primary method of payment. If the payment method was listed as Medicaid, not billed (for any reason), or self-pay, patients were considered uninsured/underinsured. Patients with a primary method of payment listed as Medicare, private/commercial insurance, other government, workers' compensation (retired 2015), blue cross/blue shield (retired 2015), or other were considered insured.

### Statistical Analysis

Through exploratory analysis of the NTDB data sets, we calculated quantitative discrete (frequencies) and continuous (time to procedure) data for each procedure and complication. Data were imported, stored, and analyzed in Jupyter notebook using Python and Microsoft Excel. Linear regression analysis was conducted in Microsoft Excel to see whether the ratio of non–Level I versus Level I trauma centers in complex fracture cases, complication rates, and uninsured/underinsured rates significantly changed over the 10 years examined. To test for a difference in time to procedures between Level I and non–Level I trauma centers each year, we performed independent Student *t*-tests using Python statistical modules (Python statistical module package statsmodels). We also used a Student *t*-test to compare trends in medians and percentages over the 10-year study period. *P* < 0.05 was considered statistically significant.

## Results

We identified in the NTDB data set a total of 301,435 patients between 18 and 65 years of age who had complex or time-sensitive fractures between 2008 and 2017. The NTDB Annual Data Bank Report is available from years 2008 to 2016 but was discontinued in 2017. In 2008, 435 centers submitted data to NTDB, of which 150 (34.5%) were Level I, and 285 were non–Level I. In 2016, 747 centers submitted data, of which 239 were Level I (32.0%), and 508 were non–Level I. Between 2008 and 2016, although there is an upward trend in the number of Level I and non–Level I centers submitting data to the NTDB, there is no significant difference in the proportion of Level I and non–Level I centers summiting these data (*P* = 0.38).

### Treatment of Complex Fractures

Table 2 shows the proportion of complex fractures recorded in the NTDB in the years 2008 to 2017 from both Level I and non–Level I trauma centers and the percent of cases done in Level I trauma centers. Regression analysis demonstrates a downward trend in the proportion of all types of complex fractures done in Level I centers (*P* < 0.05 for all but open distal femur fractures, Table 2).

**Table 2:** Trend in Proportion of Complex Orthopaedic Fractures in Level I Trauma Centers

Fracture Type	2008	2009	2010	2011	2012	2013	2014	2015	2016	2017	Percentage Slope^b^	*P* Value^b^
Proximal and distal tibia open, n (%)^a^	1127 (71%)	1204 (72%)	1221 (72%)	1274 (69%)	1397 (67%)	1361 (67%)	1472 (66%)	1716 (67%)	2036 (64%)	2144 (63%)	−0.982	**0.0001**
Proximal and distal tibia closed, n (%)^a^	5608 (65%)	5774 (64%)	6079 (64%)	7038 (66%)	7191 (64%)	7312 (63%)	8030 (61%)	8370 (62%)	8364 (59%)	8241 (62%)	−0.005	**0.018**
Distal femur open, n (%)^a^	1030 (71%)	1021 (74%)	1027 (72%)	1137 (72%)	1222 (71%)	1163 (68%)	1278 (78%)	1420 (68%)	1078 (67%)	1063 (69%)	−0.004	0.202
Distal femur closed, n (%)^a^	1989 (66%)	2212 (65%)	2385 (60%)	2656 (60%)	2757 (60%)	2830 (60%)	2915 (58%)	3049 (58%)	2258 (55%)	2216 (58%)	−0.010	**0.006**
Proximal and distal humerus open, n (%)^a^	874 (72%)	857 (70%)	869 (71%)	919 (71%)	979 (69%)	957 (66%)	1038 (69%)	1029 (65%)	989 (67%)	927 (68%)	−0.006	**0.041**
Proximal and distal humerus closed, n (%)^a^	2529 (64%)	2667 (65%)	3067 (64%)	3358 (64%)	3583 (61%)	3430 (61%)	3606 (59%)	3598 (59%)	3387 (58%)	3223 (59%)	−0.008	**0.0002**
Pelvis and acetabulum, n (%)^a^	5892 (78%)	6041 (80%)	6422 (78%)	7050 (78%)	7708 (76%)	7606 (74%)	7942 (72%)	8130 (71%)	7198 (70%)	7061 (71%)	−0.011	**0.00002**
Calcaneus closed, n (%)^a^	380 (78%)	361 (76%)	362 (75%)	451 (77%)	470 (72%)	483 (70%)	538 (69%)	675 (71%)	412 (66%)	479 (66%)	−0.014	**0.0004**
Talus closed, n (%)^a^	282 (72%)	312 (79%)	306 (78%)	391 (79%)	417 (76%)	405 (79%)	483 (72%)	540 (73%)	707 (71%)	772 (71%)	−0.007	**0.002**

a Percentage of cases done in Level I trauma centers.

b Slope and *P* value are the result of linear regression analysis.

Statistically significant *P* values (*P* < 0.05) are bolded.

**Figure 1:**
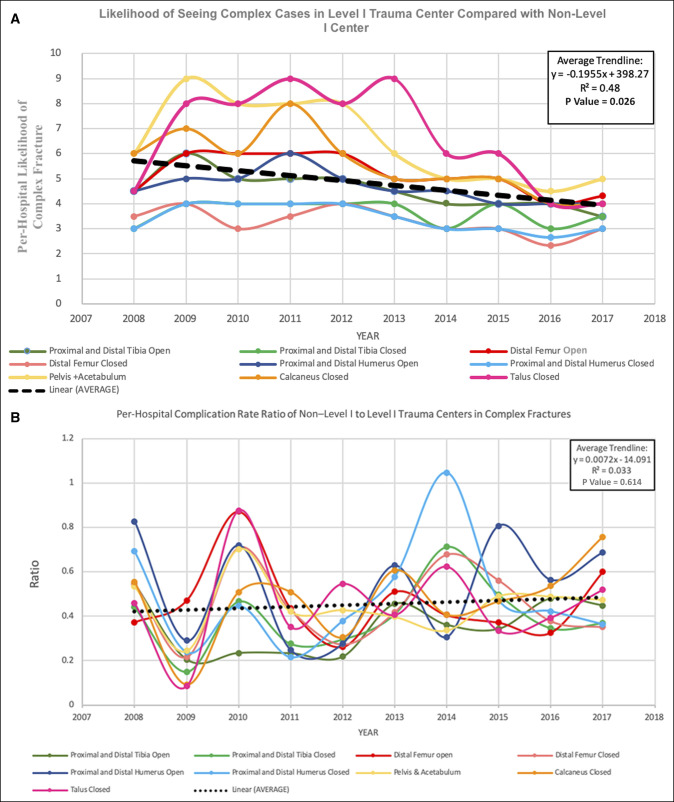
**A,** Number of cases seen in a Level I trauma centers for each seen in a Non–Level I. **B,** Per-hospital non–Level I to Level I complication rate ratio.

Figure 1, A shows the per-hospital likelihood of treating a complex fracture case in a Level I trauma center compared with a non–Level I trauma center (number of cases divided by the number of hospitals for each type). Indeed, pelvis and acetabulum fractures and closed talus fractures were nine times more likely to be seen in a Level I center than a non–Level I center between 2009 and 2012, and, moreover, Level I trauma centers were still 3 to 5 times more likely to see any of the complex fractures in 2017. However, this gap has a statistically significant downward trend over 2008 to 2017 (R^2^ = 0.48; *P* = 0.026, Figure 1, A).

### Complication Rates

Table 3 shows the percent of complication rates in Level I and non–Level I hospitals for each complex fracture and for fractures with an ISS >15. Linear regression analysis did not show significant trends in complication rates for any fracture, with the exception of a decreasing trend in closed calcaneus fracture complications at Level I trauma centers (Table 3). Moreover, when examining only extremely complex fractures—defined as fractures with ISS > 15—linear regression analysis revealed no significant trends in complication rates for any of the selected fractures, with the exception of a marginally increasing trend in open proximal and distal humerus fracture complications in non–Level I trauma centers (Table 3). Furthermore, when analyzing the entire study period, overall complication rates in Level I centers were higher than in non–Level I centers for both open proximal and distal tibia fractures (*P* = 0.010, Table 3) and closed proximal and distal tibia fractures (*P* = 0.033, Table 3). When examining only fractures with ISS > 15, Level I trauma centers had higher overall complication rates for open proximal and distal tibia fractures (*P* = 0.015, Table 3).

**Table 3:** Percent of Complication Rates in Level I and Non–Level I Trauma Centers for All Fractures and Fractures With Injury Severity Score >15

Fracture Type	Level I	2008	2009	2010	2011	2012	2013	2014	2015	2016	2017	Slope	*P* Value^b^
All fractures													
Proximal and distal tibia open	Yes	10%	12%	12%	14%	15%	13%	11%	12%	11%	11%	−0.0005	**0.010**
	No	14%	6%	6%	8%	8%	14%	7%	8%	10%	10%	0.0003	
Proximal and distal tibia closed	Yes	9%	11%	7%	9%	10%	9%	6%	7%	9%	8%	−0.002	**0.033**
	No	6%	3%	8%	6%	8%	8%	8%	8%	7%	7%	0.003	
Distal femur open	Yes	21%	18%	16%	20%	24%	22%	19%	20%	15%	11%	−0.006	0.760
	No	14%	18%	30%	20%	15%	26%	17%	16%	10%	13%	−0.008	
Distal femur closed	Yes	10%	13%	7%	11%	12%	10%	8%	9%	11%	11%	−0.0004	0.299
	No	9%	6%	11%	11%	8%	9%	11%	12%	9%	7%	0.0005	
Proximal and distal humerus open	Yes	7%	10%	8%	11%	11%	8%	11%	7%	10%	8%	0.0001	0.767
	No	10%	6%	12%	6%	7%	12%	7%	13%	11%	10%	0.003	
Proximal and distal humerus closed	Yes	6%	9%	7%	8%	7%	6%	4%	6%	7%	8%	−0.0008	0.450
	No	8%	5%	7%	4%	6%	7%	9%	6%	6%	5%	−0.0005	
Pelvis and acetabulum	Yes	13%	14%	13%	14%	15%	15%	15%	12%	14	13%	−0.0001	0.328
	No	13%	7%	20%	13%	15%	13%	10%	13%	13%	12%	−0.0008	
Calcaneus closed	Yes	10%	15%	13%	11%	14%	10%	11%	12%	5%	6%	−0.007^a^	0.545
	No	10%	3%	15%	13%	10%	14%	9%	12%	6%	5%	−0.003	
Talus closed	Yes	15.%	19%	10%	12%	9%	12%	10%	13%	9%	10%	−0.006	0.253
	No	12%	3%	18%	10%	11%	11%	13%	10%	5%	6%	−0.004	
Fractures with injury severity score>15													
Proximal and distal tibia open	Yes	6.5%	7.2%	7.6%	8.0%	9.2%	6.8%	6.3%	8.8%	7.1%	7.4%	0.0003	**0.015**
	No	7.1%	4.5%	1.5%	2.5%	4.4%	9.7%	4.4%	4.4%	6.3%	7.6%	0.0026	
Proximal and distal tibia closed	Yes	5.6%	5.3%	4.3%	5.1%	3.9%	2.1%	2.0%	3.7%	5.3%	4.5%	−0.0015	0.158
	No	1.8%	1.6%	2.7%	1.5%	4.8%	6.8%	3.5%	3.5%	3.0%	3.0%	0.0019	
Distal femur open	Yes	12.5%	12.9%	10.4%	14.1%	14.8%	13.0%	13.0%	13.4%	11.3%	8.0%	−0.0026	0.269
	No	9.8%	8.2%	14.3%	9.3%	12.4%	18.6%	10.5%	10.5%	7.0%	8.5%	−0.0018	
Distal femur closed	Yes	6.2%	7.5%	5.2%	7.5%	4.8%	2.8%	2.1%	5.9%	7.0%	7.1%	−0.0005	0.293
	No	3.3%	3.3%	4.1%	3.6%	6.4%	8.4%	5.3%	5.3%	4.3%	3.6%	0.0014	
Proximal and distal humerus open	Yes	5.9%	8.0%	5.8%	8.1%	6.1%	6.5%	7.1%	5.5%	7.4%	5.7%	−0.0006	0.300
	No	3.3%	3.1%	6.0%	2.7%	5.6%	7.3%	7.5%	7.5%	7.0%	8.4%	0.0059^a^	
Proximal and distal humerus closed	Yes	5.0%	6.3%	4.8%	4.3%	3.6%	2.4%	1.4%	4.4%	5.0%	5.2%	−0.0011	0.400
	No	5.2%	2.9%	2.1%	2.9%	4.8%	6.1%	3.1%	3.1%	3.6%	3.4%	−0.0003	
Pelvis and acetabulum	Yes	9.7%	10.3%	9.6%	9.1%	10.0%	9.5%	9.6%	9.2%	10.3%	10.0%	0.0019	0.844
	No	8.8%	4.6%	9.4%	11.4%	13.5%	10.9%	8.9%	8.9%	9.9%	9.5%	0.00008	
Calcaneus closed	Yes	9.2%	12.1%	7.4%	7.5%	6.8%	9.4%	6.6%	9.2%	5.5%	7.3%	−0.0033	0.070
	No	7.1%	2.3%	8.7%	4.9%	9.2%	9.8%	6.0%	6.0%	2.1%	4.9%	−0.0018	
Talus closed	Yes	5.9%	11.4%	10.0%	7.5%	11.9%	7.5%	5.0%	8.6%	3.4%	3.8%	−0.0057	0.467
	No	7.6%	0.0%	4.5%	12.0%	10.1%	9.5%	6.2%	6.2%	4.3%	3.6%	−0.0009	

a Indicates a slope with *P* value <0.05.

b *P* value for comparing percent complication in Level I and non–Level I throughout the study period.

Statistically significant *P* values (*P* < 0.05) are bolded.

Figure 1, B shows the per-hospital ratio of complication rates for non–Level I trauma centers versus Level I trauma centers for each complex fracture. Aside from proximal humerus fractures in 2014, per-hospital complication rates were consistently lower in non–Level I trauma centers compared with Level I trauma centers with no observable trend in the study period (R^2^ = 0.033; *P* = 0.614).

### Time-Sensitive Fractures

**Figure 2:**
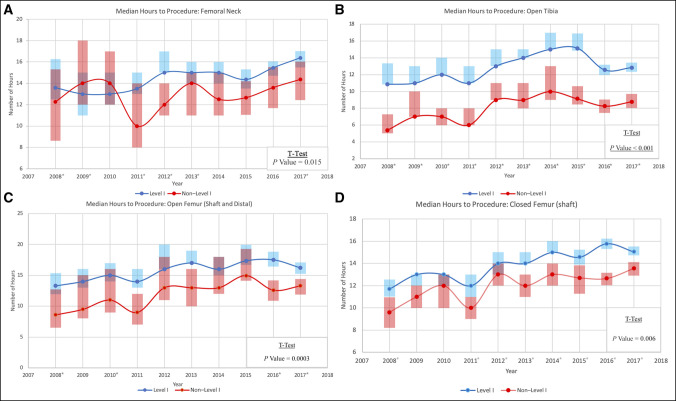
**A**–**D,** Median hours to surgical procedure for each time-sensitive fracture. **A,** Femoral neck, (**B**) open tibia, (**C**) open femur, and (**D**) closed femur. The shaded blocks indicate the 95% confidence interval. The star (*) located next to the year indicates that there is a statistically significant difference between the median hour values of Level I and non–Level I trauma centers for that year.

Figure 2, A–D displays the median number of hours to the beginning of the surgical procedure for each of the four time-sensitive fractures seen at Level I and non–Level I trauma centers. The median time to procedure in non–Level I trauma centers was consistently less than that of Level I trauma centers with the exception of 2009 and 2010 in femoral neck fractures (Figure 2, A–D). A *t*-test of the median values for each year in the study period was used to examine the overall difference and revealed a statistically significant gap in the median time to procedure between Level I and non–Level I trauma centers for all time-sensitive procedures (femoral neck, *P* = 0.015; open tibia, *P* < 0.001; open femur [shaft and distal], *P* = 0.0003; closed femur [shaft], *P* = 0.006) (Figure 2, A–D).

### Health Insurance Status

**Figure 3:**
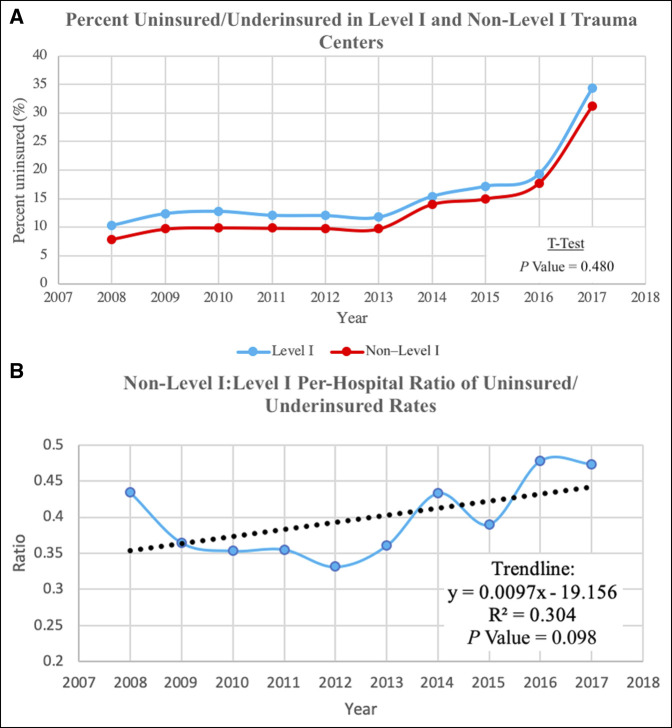
**A,** Rate of uninsured/underinsured patients in Level I and non–Level I trauma centers. **B,** Per-hospital ratio of percent of uninsured/underinsured patients in non–Level I vs. Level I trauma centers.

There were a total of 47,891 uninsured/underinsured patients in Level I and non–Level I trauma centers. For the entire 10-year period, non–Level I trauma centers had a lower rate of uninsured/underinsured patients (Figure 3, A). The uninsured/underinsured patient rate was relatively constant in trauma centers of all levels from 2008 to 2016 (Figure 3, A). In 2008 to 2013, there was approximately a 5% difference between Level I and non–Level I trauma centers in the percent of uninsured/underinsured patients, although the gap narrowed in 2014 to 2017 (Figure 3, A). In 2017, the rate of uninsured/underinsured patients recorded in the NTDB nearly doubled in both Level I and non–Level I hospitals. Overall, however, there were no significant differences in the percentage of uninsured/underinsured patients treated in Level I and non–Level I centers throughout the study period (*P* = 0.480, Figure 3, A). Moreover, although the per-hospital percentage of uninsured/underinsured patients was consistently higher in Level I hospitals, there were no statistically significant trends in the study period (*P* = 0.098, Figure 3, B).

## Discussion

This study has shown a steady decrease in the proportion of reported complex orthopaedic trauma cases treated in Level I trauma centers between 2008 and 2017. The increase in the management of complex orthopaedic trauma in non–Level I centers does not appear to be associated with worsening patient outcomes as measured by complication rates and time to treatment of time-sensitive orthopaedic trauma. Furthermore, this study demonstrated that the observed trends do not seem to be influenced by the payer mix.

In recent years, the number of orthopaedic trauma fellowship positions offered and filled has increased steadily, whereas the number of academic positions in Level I trauma centers has remained constant or decreased.^5^ A logical result of this trend is that more fellowship-trained orthopaedic trauma surgeons are working in non–Level I centers. Our study demonstrated a steady decrease in the relative proportion of complex trauma cases performed in Level I centers. This finding may be a testament to non–Level I trauma centers' growing ability in managing these injuries. A recent study by Sielatycki et al^6^ demonstrated that the number of pelvis and acetabulum fractures performed in individual trauma centers has decreased in the past decade. The authors rightfully raise the concern that the lowered volume of cases may lead to lower expertise in managing these complex cases based on experience from the hip fracture, arthroplasty, and spine literature.^9,10,12,13^ Although not required by the ACS, resident training is being performed in Level II and III centers. It is hard to determine the effect that this shift in treatment patterns of complex trauma may have on training quality. However, it does warrant consideration of using local non–Level I centers for training if they are receiving larger proportions of the complex trauma.

Several studies have previously demonstrated the benefits of Level I trauma centers for nonorthopaedic injuries.^1-3,14^ Other studies, however, have questioned these results.^15-17^ Alkhoury et al^15^ used the NTDB between 2001 and 2006 to assess patients with an isolated traumatic head injury. In their study, Level II centers were not significantly inferior to Level I trauma centers regarding outcomes and complication rate. Another NTDB study on patients with blunt and penetrating trauma with an ISS ≥15 showed no correlation between higher trauma center volumes and improved survival.^16^ In a recent study, that compared Level I and II trauma centers with nontrauma centers, the authors focused on 829 adult patients (from 18 trauma centers) with pelvic trauma.^18^ Patients who had at least one pelvic ring or acetabulum injury were evaluated for complications and function at up to 1 year. The authors found reduced mortality rates during the in-hospital stay, within 90 days, and within 1 year for severe acetabulum fracture (AO/OTA 62-B and 62-C); reduced mortality within 90 days and within 1 year for combined pelvis ring and acetabulum injuries; and reduced mortality within 1 year for unstable pelvic ring injury (AO/OTA 61-B and 61-C). The current study did not combine Level I and Level II together, which can explain the lack of any increase in complications in non–Level I centers for pelvis and acetabulum fractures and any other type of complex fracture. Another reason for this discrepancy may be the lack of accurate subclassification of pelvis and acetabulum fractures in the NTDB. It is noteworthy that the complication rates in non–Level I centers were lower than Level I centers after controlling for ISS, particularly for open proximal and distal tibia fractures. Despite this, patient selection bias may be the reason for the increased complication rate at Level I hospitals. However, this study cannot support the hypothesis that treatment in non–Level I centers is compromising patient care.

There is no clinical study support for the six-hour rule for the treatment of open fractures.^19-21^ However, there is a consensus that these fractures need treatment as soon as possible if an appropriate level of care is available.^14,19^ Other injuries, such as femoral shaft fracture and femoral neck fractures in young patients, should be treated as soon as possible.^22,23^ This study demonstrated that open tibia fractures, femoral shaft fractures, and femoral neck fractures were not treated earlier in Level I hospitals compared with non–Level I counterparts. Using the NTDB, Namdari et al^14^ found that patients presenting to Level I trauma centers with open tibia fractures were more likely to undergo surgical intervention after 6 hours or with a 24-hour delay. Moreover, in a study of 204 patients admitted to Level I and 1425 patients admitted to Level II trauma centers between January 2008 and December 2012 for isolated hip fracture, Van Larrhoven et al^24^ found that a higher percentage (85% versus 65%) of patients with hip fractures were treated within 1 day of arrival in Level II hospitals compared with Level I hospitals. The current study shows similar delays in treating time-sensitive fractures in Level I trauma centers compared with non–Level I trauma centers. These findings may suggest an improved level of care in non–Level I centers compared with Level I centers for time-sensitive fractures.

Previous studies demonstrated that insurance status is an independent predictor for transferring patients with trauma from Level III/IV to Level I centers.^25^ Twelve percent of 2008 patients evaluated at Level III/IV hospital were transferred to Level I hospitals. Patients without commercial health insurance had a 2.4 (95% confidence interval: 1.6 to 3.6) times greater chance of being transferred than patients with commercial insurance after controlling for injury severity, age, and sex in a multivariable analysis. Other studies have shown that Level I centers treat a disproportionate amount of uninsured patients.^26-28^ The current study payer mix (insurance status) in both level I and non–Level I centers remained stable throughout 2008 to 2017. Both Level I and non–Level I trauma centers had a similar increase in uninsured/underinsured patients in 2017, and the increase in uninsured/underinsured patients in 2017 for both Level I and non–Level I centers may have been a direct result of Medicaid expansion during the study period. Thus, our findings suggest that the payer mix is not responsible for the increasing complex trauma treated in non–Level I centers.

Our study's strengths lay in the large study population and the national representation afforded by the NTDB. This study, however, also has some notable limitations. The NTDB is not a population-based study, but the database is a voluntary registry of hospitals with observed variation across reporting practices. An important limitation of this study is the lack of multivariable analysis to control for confounding factors that can influence the rate of complications and surgical time. Therefore, this study's results do not show that a designation of Level I trauma center is independently associated with increased complication rates of complex fractures and increased time to treatment of time-sensitive fractures. Moreover, because the NTDB only records inpatient complications, the analysis of complications was limited to acute complications. This study aimed to report trends of care of complex orthopaedic trauma between 2008 and 2017, not the comparison of quality of care in Level I and non–Level I centers. Simply stated, the results of this study only show that there is a decline in the proportion of complex trauma cases done in Level I centers, without a concomitant increase in reported complications in non–Level I centers, or a decreased quality of care as manifested by the time to surgery of time-sensitive procedures in non–Level I centers. In addition, because having either orthopaedic or nonorthopaedic complications was reported as a single binary variable, trends and differences in each type of complication rate may have been missed. Of note, the NTDB registry does not record information on fracture classification, fragmentation, or bone loss, so it is possible that more complex fracture patterns were treated in Level I centers. However, to mitigate such concerns, patients were matched by ISS and insurance status during analysis (Table 3). Finally, although the NTDB reports the percent of hospitals submitting to the registry by state, it does not stratify this percent by Level I and non–Level I hospitals to determine the percent of Level I and non–Level I hospitals that submit data to the NTDB, which may skew results.

In summary, by analyzing consecutive data sets in the NTDB, this study describes national practice trends and shows a reduction of complex trauma treatment in Level I trauma centers, without demonstrating an adverse effect on patient outcome. These data may indicate an increasing level of expertise in managing complex musculoskeletal injury outside of Level I trauma centers. The dispersal of complex fracture care may lower the volume of complex cases for individual surgeons, making it difficult for young surgeons to reach the necessary surgical skills if their training is limited to Level I centers. Policymakers should notice this trend to ensure the continued quality of orthopaedic trauma training and maintenance of expertise in the management of complex fractures.

## Data Accessibility

Supplemental information such as study protocol, raw data, or programming code can be provided on reasonable written request to the corresponding author.
